# Prediction of longitudinal clinical outcomes after acute myocardial infarction using a dynamic machine learning algorithm

**DOI:** 10.3389/fcvm.2024.1340022

**Published:** 2024-04-05

**Authors:** Joo Hee Jeong, Kwang-Sig Lee, Seong-Mi Park, So Ree Kim, Mi-Na Kim, Shung Chull Chae, Seung-Ho Hur, In Whan Seong, Seok Kyu Oh, Tae Hoon Ahn, Myung Ho Jeong

**Affiliations:** ^1^ Division of Cardiology, Department of Internal Medicine, Anam Hospital, Korea University Medicine, Seoul, Republic of Korea; ^2^ Korea University College of Medicine, AI Center, Anam Hospital, Seoul, Republic of Korea; ^3^ Department of Internal Medicine, Kyungpook National University Hospital, Daegu, Republic of Korea; ^4^ Cardiovascular Medicine, Keimyung University Dongsan Medical Center, Daegu, Republic of Korea; ^5^ Department of Internal Medicine, Chungnam National University Hospital, Chungnam National University, College of Medicine, Daejeon, Republic of Korea; ^6^ Division of Cardiology, Department of Internal Medicine, Wonkwang University School of Medicine, Iksan, Republic of Korea; ^7^ Department of Cardiology, Na-eun Hospital, Incheon, Republic of Korea; ^8^ Department of Cardiology, Chonnam National University Hospital, Gwangju, Republic of Korea

**Keywords:** body mass index, machine learning analysis, myocardial infarction, artificial intelligence, prediction model

## Abstract

Several regression-based models for predicting outcomes after acute myocardial infarction (AMI) have been developed. However, prediction models that encompass diverse patient-related factors over time are limited. This study aimed to develop a machine learning-based model to predict longitudinal outcomes after AMI. This study was based on a nationwide prospective registry of AMI in Korea (*n* = 13,104). Seventy-seven predictor candidates from prehospitalization to 1 year of follow-up were included, and six machine learning approaches were analyzed. Primary outcome was defined as 1-year all-cause death. Secondary outcomes included all-cause deaths, cardiovascular deaths, and major adverse cardiovascular event (MACE) at the 1-year and 3-year follow-ups. Random forest resulted best performance in predicting the primary outcome, exhibiting a 99.6% accuracy along with an area under the receiver-operating characteristic curve of 0.874. Top 10 predictors for the primary outcome included peak troponin-I (variable importance value = 0.048), in-hospital duration (0.047), total cholesterol (0.047), maintenance of antiplatelet at 1 year (0.045), coronary lesion classification (0.043), N-terminal pro-brain natriuretic peptide levels (0.039), body mass index (BMI) (0.037), door-to-balloon time (0.035), vascular approach (0.033), and use of glycoprotein IIb/IIIa inhibitor (0.032). Notably, BMI was identified as one of the most important predictors of major outcomes after AMI. BMI revealed distinct effects on each outcome, highlighting a U-shaped influence on 1-year and 3-year MACE and 3-year all-cause death. Diverse time-dependent variables from prehospitalization to the postdischarge period influenced the major outcomes after AMI. Understanding the complexity and dynamic associations of risk factors may facilitate clinical interventions in patients with AMI.

## 1 Introduction

Cardiovascular disease is a major health burden globally, resulting in approximately 17 million deaths annually. It also contributes to 10% of the global disease burden (1, 2). Ischemic heart disease is the single most common cause of cardiovascular death and overall mortality worldwide (3). Interventional strategies for ischemic heart disease have advanced in the past three decades, and the associated clinical outcomes and mortality rates have improved. However, the prognosis of ischemic heart disease is variable, encompassing a variety of diverse risk factors (4–6). The appropriate prediction of prognosis in acute myocardial infarction (AMI) is crucial for proper clinical decision-making at an individual patient level. Appropriate prediction is important for the alleviation of expenditures and effective distribution of resources. Over the last two decades, several prognosis predicting models for AMI patients have been introduced, which reflect numerous patient-level risk factors (7, 8). However, a systematic predictive model that comprehends various risk factors across different time periods—namely prehospitalization variables, in-hospital events, procedural results, and postdischarge variables—is lacking. This is especially true in the prediction of long-term outcomes following AMI events.

Machine learning identifies the underlying patterns from large volumes of datasets and can facilitate effective predictions (9–11). Machine learning-based algorithms have led to the development of prediction models that integrate multiple patient-related variables. However, conflicting results have been reported regarding the efficacy of machine learning-based algorithms in predicting clinical outcomes after cardiovascular events (12, 13). Therefore, this study aimed to develop a machine learning-based prediction model that estimates longitudinal clinical outcomes, including mortality and major adverse cardiovascular event (MACE), after AMI. The study also investigated the performance of various machine learning-based algorithms.

## 2 Materials and methods

### 2.1 Participants and variables

This study was based on a nationwide, prospective, observational registry of AMI in South Korea (KAMIR-NIH). The KAMIR-NIH registry was established across 20 tertiary centers equipped for coronary interventions, spanning from November 2011–December 2015 (14). The KAMIR-NIH registry consists of 13,104 patients with AMI and their 3-year follow-up clinical outcomes. The protocols of the KAMIR-NIH conformed to the principles of the Declaration of Helsinki and were approved by the institutional review boards of each center (CNUH-2011-172). Written informed consent was obtained from each patient or legal representative. All data were collected through electronic case report forms in a data management system established by the Centers for Disease Control and Prevention, Ministry of Health and Welfare, Republic of Korea (iCReaT Study No. C110016; KCT-0000863). A detailed protocol and additional information about data collection for the KAMIR-NIH registry have been previously published (14, 15).

The primary outcome was defined as all-cause death at 1 year of follow-up. The secondary outcomes included five dependent variables: (1) cardiovascular death at the 1-year follow-up, (2) MACE at the 1-year follow-up, (3) all-cause death at the 3-year follow-up, (4) cardiovascular death at the 3-year follow-up, and (5) MACE at the 3-year follow-up. The 3-year outcomes were confined to any events that occurred by the 3-year follow-up without occurring at the 1-year follow-up. Death was classified as cardiovascular in origin if no other non-cardiac causes were identified. MACE was defined as a composite outcome requiring clinical intervention, including myocardial infarction, repeat percutaneous coronary intervention (PCI) of target or non-target vessel revascularization, coronary artery bypass graft, stent thrombosis, cerebrovascular accident (ischemic stroke or hemorrhagic stroke), and re-hospitalization due to heart failure aggravation. Seventy-seven independent variables were included to predict outcomes derived from various time periods: prehospitalization, in-hospitalization, during coronary intervention, and postdischarge (Supplementary Table S1). Data for predicting outcomes in the candidates comprised demographic, health, and treatment information, including procedural factors such as coronary angiography and PCI.

### 2.2 Statistical analysis

Six machine learning approaches (artificial neural network, decision tree, logistic regression, naïve Bayes, random forest, and support vector machine) were used to predict all-cause death, cardiovascular death, and MACE at the 1-year and 3-year follow-ups (16–19). The limited-memory Broyden–Fletcher–Goldfarb–Shanno algorithm was used for the optimization of the artificial neural network, and the number of neurons was 10 for each of its two hidden layers. The random forest employed 1,000 trees without imposing a maximum depth.

A decision tree comprises three elements: a test on an independent variable (intermediate note), an outcome of the test (branch), and the value of the dependent variable (terminal node). A naïve Bayesian classifier performs classification based on Bayes' theorem. In contrast, a random forest is a collection of many decision trees, employing majority votes on the dependent variable (bootstrap aggregation) (16). Meanwhile, a support vector machine estimates a group of support vectors, forming a line or space known as a hyperplane. This hyperplane effectively separates data by maximizing the gap between various subgroups. Lastly, an artificial neural network consists of neurons, which are information units interconnected through weights (16).

Data on 9,661 observations with complete information were randomly divided into training and validation sets using a 75:25 ratio (7,246 vs. 2,415). Accuracy, which quantifies the ratio of correct predictions among the 2,415 observations, was employed as a standard for validating the models. The area under the receiver-operating-characteristic curve (AUC) was the other performance indicator in this study. Sensitivity, specificity, and the F1 score were taken into consideration to enhance the understanding of accuracy. Random forest variable importance, defined as the contribution of a certain variable to the performance of the random forest, was used to examine the major predictors of the six dependent variables.

Further analysis of SHapley Additive exPlanations (SHAP) values was adopted to illustrate the relative attribution of independent variables to each outcome. The SHAP value of a predictor for a participant measures the difference between what the random forest predicts for the probability of the death/event with and without the predictor. These are described as SHAP summary plots. The SHAP dependence plot for a predictor registers a relationship between the value of the predictor and its SHAP value.

For rigorous analysis, the random split and examination were iterated 50 times. The ensuing average was then employed for external validation (18, 19). Python (CreateSpace: Scotts Valley, 2009) was employed for the analysis between December 15, 2021, and May 15, 2022.

## 3 Results

**Figure 1:**
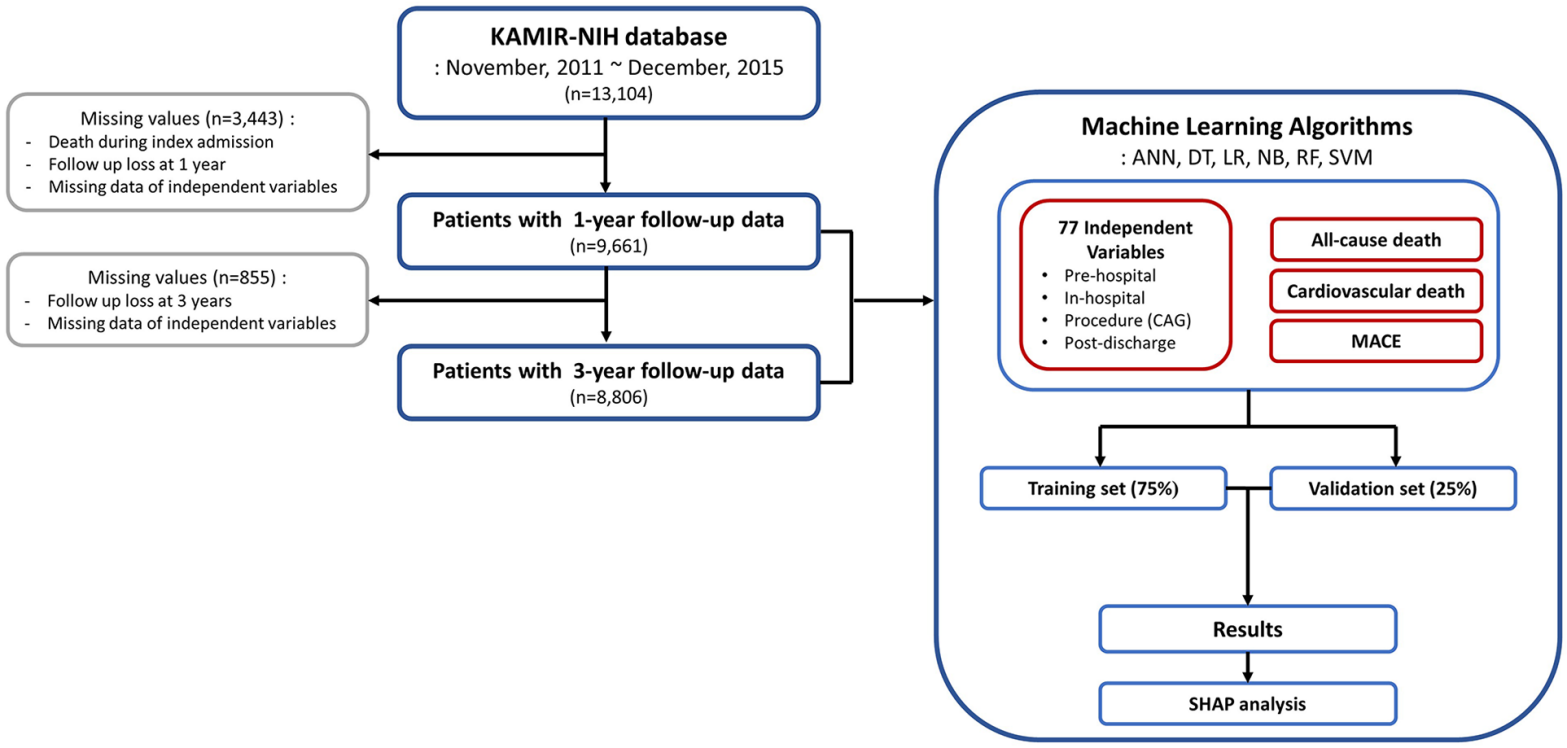
Flowchart of the study process. KAMIR-NIH, Korean Acute Myocardial Infarction-National Institutes of Health; ANN, artificial neural network; DT, decision tree; LR, logistic regression; NB, naïve Bayes; RF, random forest; SVM, support vector machine; CAG, coronary angiography; MACE, major adverse cardiovascular event; SHAP, shapley additive explanations.

A total of 13,104 patients were enrolled in the KAMIR-NIH registry, and from this cohort, 9,661 patients with data on dependent and independent variables were further selected (Figure 1). Among them, 8,806 patients with 3-year follow-up data were analyzed for the 3-year outcome prediction. For the 9,661 patients included in the 1-year outcome analysis, the mean age of onset was 62.6 ± 12.2 years and 2,297 (23.8%) were female (Table 1). ST-elevation myocardial infarction was diagnosed in 4,975 patients (51.5%). Cardiogenic shock was diagnosed in 576 (6.0%) patients, and extracorporeal membrane oxygenation was applied in 24 patients (0.2%). With respect to procedures, 9,016 patients (93.3%) underwent PCI, whereas 612 patients (6.3%) received balloon angioplasty only (patients without coronary angiography data were excluded from the analysis). Similar clinical characteristics were observed in the study population included in the 3-year outcome analysis (Supplementary Table S2). During the 1-year follow-up, 35 patients (0.4%) died, with 20 (0.2%) deaths attributed to cardiovascular causes. Moreover, MACE occurred in 314 patients (3.3%). During the 3-year follow-up, 144 patients (1.6%) died, with 79 (0.9%) classified as cardiovascular deaths, and MACE occurred in 254 patients (2.9%, Supplementary Table S2).

**Table 1:** Baseline characteristics of the study participants in the 1-year outcome analysis.

Variables	Total (*n* = 9,661)
Prehospital variables	
Age, years	62.6 ± 12.2
Female sex	2,297 (23.8)
Body mass index (kg/m^2^)	24.1 ± 3.2
Smoking status	
Current smoker	4,085 (42.3)
Hypertension	4,727 (48.9)
Diabetes mellitus	2,580 (26.7)
HbA1c (%)	6.3 ± 1.2
Use of insulin	204 (2.1)
Dyslipidemia	1,125 (11.6)
Baseline low-density lipoprotein level (mg/dl)	113.8 ± 36.9
Baseline high-density lipoprotein level (mg/dl)	42.6 ± 11.1
Baseline triglyceride level (mg/dl)	134.4 ± 110.6
Baseline total cholesterol level (mg/dl)	180.7 ± 44.0
Previous history of myocardial infarction or angina	1,333 (13.8)
Previous history of PCI or CABG	974 (10.1)
Previous history of heart failure	92 (1.0)
Previous history of cerebrovascular accident	570 (5.9)
Family history of coronary artery disease	669 (6.9)
In-hospital variables	
Typical chest pain	8,634 (89.4)
Dyspnea	1,991 (20.6)
Killip class	
I	7,949 (82.3)
II	757 (7.8)
III	555 (5.7)
IV	400 (4.1)
STEMI	4,975 (51.5)
Anterior myocardial infarction	3,710 (38.4)
Atrioventricular block (2nd degree or more)	82 (0.8)
Atrial fibrillation	270 (2.8)
Ventricular tachycardia or ventricular fibrillation	367 (3.8)
New-onset heart failure	295 (3.1)
Initial cardiac arrest	447 (4.6)
Cardiogenic shock	576 (6.0)
Use of extracorporeal membrane oxygenation	24 (0.2)
Systolic blood pressure (mmHg)	131.2 ± 28.4
Diastolic blood pressure (mmHg)	79.5 ± 17.4
Heart rate	77.4 ± 18.1
Peak creatine-kinase myoglobin level (ng/ml)	112.8 ± 143.1
Peak troponin-I level (ng/ml)	43.8 ± 99.2
NT-proBNP level (pg/ml)	1,152.4 ± 5,003.7
Hemoglobin level (g/dl)	14.0 ± 1.9
Creatinine level (mg/dl)	1.0 ± 0.9
C-reactive protein level (mg/L)	0.8 ± 4.3
Left ventricular ejection fraction (%)	52.6 ± 10.3
Regional wall motion index	1.3 ± 0.3
In-hospital duration (d)	5.0 ± 3.2
Procedural variables	
Onset-to-door time (h)	23.5 ± 124.4
Door-to-balloon time (h)	19.5 ± 408.9
Onset-to-balloon time (h)	43.1 ± 428.6
Vascular approach (transfemoral/radial	
Transfemoral	5,880 (60.9)
Transradial	3,670 (38.0)
Both	111 (1.1)
Culprit lesion	
Left anterior descending	4,527 (46.9)
Left circumflex	1,687 (17.5)
Right coronary artery	3,270 (33.8)
Left main	177 (1.8)
Coronary lesion classification	
A	127 (1.3)
B1	1,132 (11.7)
B2	3,660 (37.9)
C	4,742 (49.1)
Initial TIMI flow of culprit lesion	
0	4,488 (46.5)
1	1,072 (11.1)
2	1,508 (15.6)
3	2,593 (26.8)
Post-PCI TIMI	
0	23 (0.2)
1	24 (0.2)
2	227 (2.3)
3	9,387 (97.2)
Stent or balloon	
None	33 (0.3)
Stent	9,016 (93.3)
Balloon	612 (6.3)
Number of stents	1.3 ± 0.8
Total stent length (mm)	27.5 ± 15.4
Stent generation	3.1 ± 0.4
Bare metal stent	645 (6.7)
First generation	228 (2.4)
Second generation	814 (8.4)
Staged PCI	7,974 (82.5)
Thrombus aspiration	941 (9.7)
Use of glycoprotein IIb/IIIa inhibitor	2,422 (25.1)
Use of intra-aortic balloon pump	1,509 (15.6)
Use of IVUS or OCT	196 (2.0)
Post-discharge variables	2,156 (22.3)
P2Y12 inhibitor at discharge	
None	81 (0.8)
Clopidogrel	6,545 (67.7)
Prasugrel	1,092 (11.3)
Ticagrelor	1,943 (20.1)
Beta-blocker at discharge	8,403 (87.0)
Renin-angiotensin system inhibitor at discharge	8,004 (82.8)
Statin use at discharge	9,215 (95.4)
Use of high-intensity statin at discharge	2,759 (28.6)
MACE at 6 months	343 (3.6)
Maintenance of antiplatelet at 12 months	
Dual antiplatelet	6,890 (71.3)
Mono antiplatelet	2,615 (27.1)
No antiplatelet	156 (1.6)
Maintenance of beta-blocker at 12 months	7,672 (79.4)
Maintenance of renin-angiotensin system inhibitor at 12 months	7,215 (74.7)
Maintenance of statin at 12 months	9,042 (93.6)
Outcomes	
All-cause death	35 (0.4)
Cardiovascular death	20 (0.2)
MACE	314 (3.3)

NT-proBNP, N- terminal brain natriuretic peptide; PCI, percutaneous coronary intervention; CABG, coronary artery bypass graft; STEMI, ST-elevation myocardial infarction; MACE, major adverse cardiovascular event; TIMI, thrombolysis in myocardial infarction; IVUS, intravascular ultrasound; OCT, optical coherence tomography.

**Figure 2:**
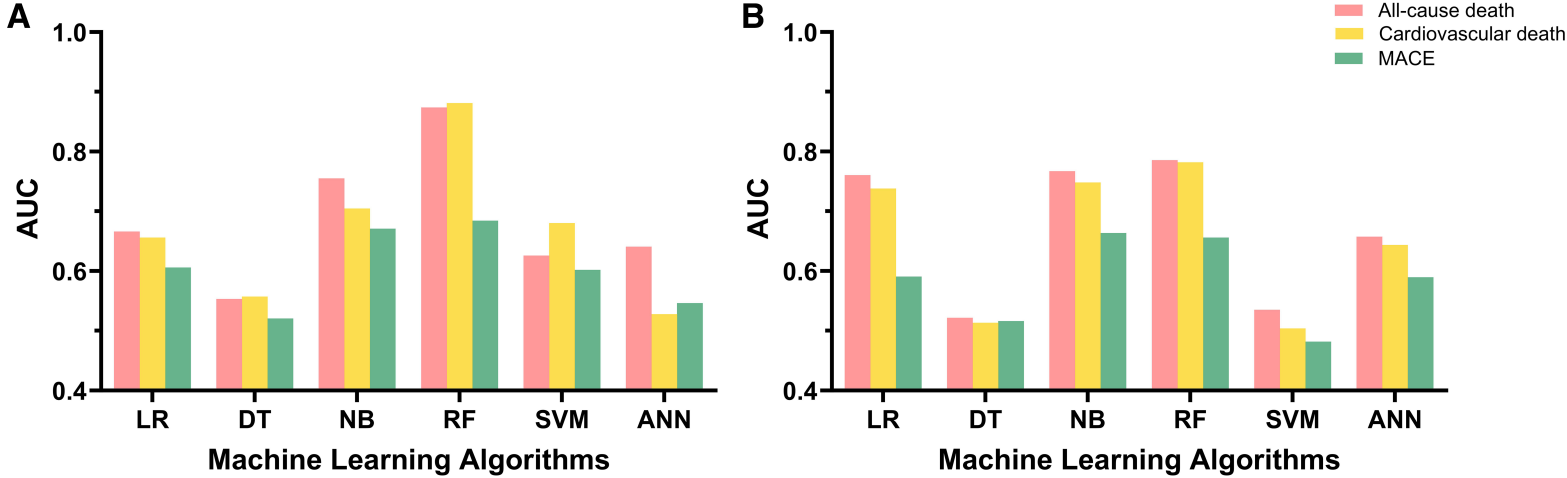
Model performance of machine learning algorithms and logistic regression. (**A**) Model performance for 1-year follow-up data. (**B**) Model performance for 3-year follow-up data. AUC, area under the receiver-operating characteristic curve; LR, logistic regression; DT, decision tree; NB, naïve Bayes; RF, random forest; SVM, support vector machine; ANN, artificial neural network; MACE, major adverse cardiovascular event.

For the primary outcome, the random forest algorithm showed best performance, achieving an AUC of 0.874 and an accuracy of 99.6% (Figure 2, Table 2, Supplementary Figure S1). The consistent excellence of the random forest algorithm carried over to the secondary outcomes, surpassing the performance of logistic regression.

**Table 2:** Model performance of machine learning algorithms and logistic regression.

Outcome	1-year		3-year	
	Accuracy	AUC	Accuracy	AUC
Cardiovascular Death				
Model				
Logistic Regression	0.9976	0.6562	0.9908	0.7381
Decision Tree	0.9956	0.5575	0.9797	0.5134
Naïve Bayes	0.9205	0.7048	0.8478	0.7481
Random Forest	0.9977	0.8811	0.9909	0.7820
Support Vector Machine	0.9975	0.6803	0.9909	0.5041
Artificial Neural Network	0.9976	0.5279	0.9907	0.6439
All-Cause Death				
Model				
Logistic Regression	0.9960	0.6664	0.9827	0.7606
Decision Tree	0.9924	0.5532	0.9642	0.5219
Naïve Bayes	0.8827	0.7552	0.8247	0.7670
Random Forest	0.9962	0.8738	0.9830	0.7857
Support Vector Machine	0.9967	0.6258	0.9830	0.5352
Artificial Neural Network	0.9961	0.6409	0.9826	0.6576
MACE				
Model				
Logistic Regression	0.9670	0.6059	0.9712	0.5907
Decision Tree	0.9304	0.5208	0.9375	0.5163
Naïve Bayes	0.8074	0.6708	0.8412	0.6636
Random Forest	0.9671	0.6843	0.9712	0.6560
Support Vector Machine	0.9673	0.6019	0.9712	0.4818
Artificial Neural Network	0.9666	0.5466	0.9710	0.5896

AUC, area under the curve; MACE, major adverse cardiovascular event.

**Figure 3:**
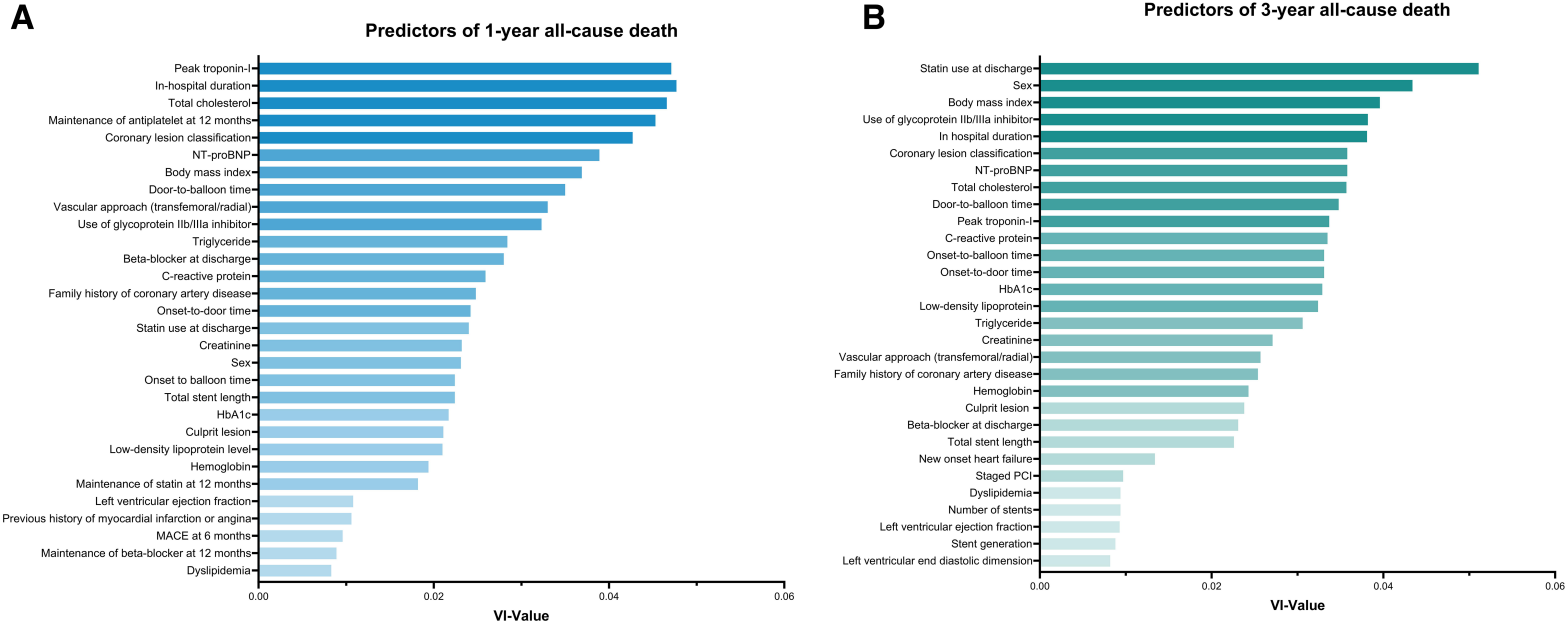
Predictors for 1-year and 3-year all-cause death as determined by random forest analysis. (**A**) Top 30 predictors for 1-year all-cause death based on random forest variable importance. (**B**) Top 30 predictors for 3-year all-cause death based on random forest variable importance. NT-proBNP, N-terminal pro-brain natriuretic peptide; MACE, major adverse cardiovascular event; PCI, percutaneous coronary intervention; VI, variable importance.

Identified predictors of 1-year all-cause death included peak troponin-I (variable importance value = 0.048), in-hospital duration (0.047), total cholesterol (0.047), maintenance of antiplatelet at 1 year (0.045), coronary lesion classification (0.043), N-terminal pro-brain natriuretic peptide levels (0.039), body mass index (BMI) (0.037), door-to-balloon time (0.035), vascular approach (0.033), and use of glycoprotein IIb/IIIa inhibitor (0.032, Figure 3A). For the 3-year follow-up, predictors of all-cause death included statin use at discharge (0.051), sex (0.043), BMI (0.040), use of glycoprotein IIb/IIIa inhibitor (0.038), in-hospital duration (0.038), NT-proBNP (0.036), coronary lesion classification (0.036), total cholesterol (0.036), door-to-balloon time (0.035), and peak troponin-I (0.034, Figure 3B). The importance ranking of BMI stayed within the top seven predictors for all outcomes, except for the prediction of 3-year MACE, where it dropped to the 14th position. Further details based on the random forest algorithm are provided in Supplementary Tables 3, 4.

**Figure 4:**
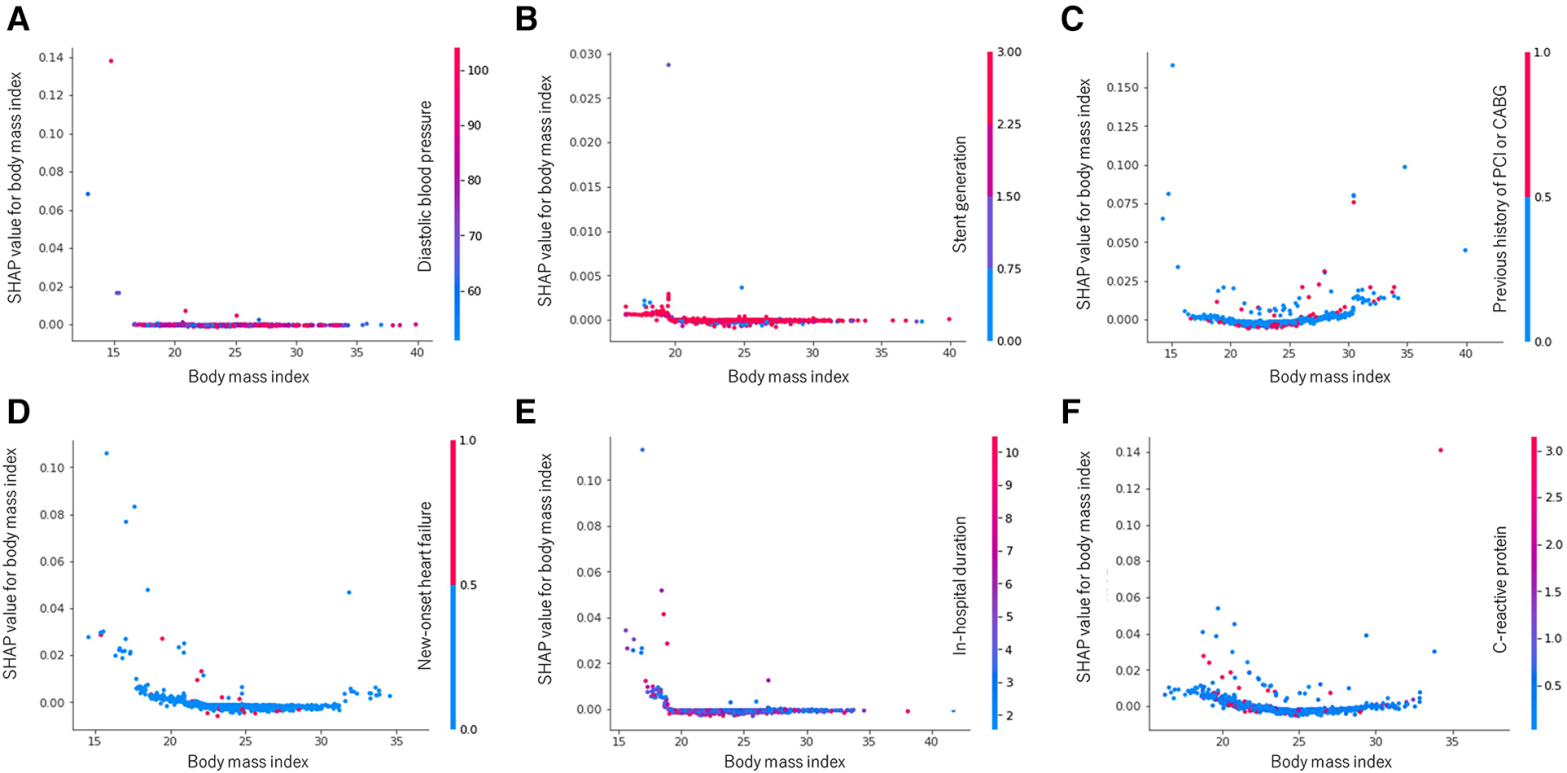
Random forest SHAP dependence plots: body mass index. (**A**) All-cause death at 1 year. (**B**) Cardiovascular death at 1 year. (**C**) MACE at 1 year. (**D**) All-cause death at 3 years. (**E**) Cardiovascular death at 3 years. (**F**) MACE at 3 years. MACE, major adverse cardiovascular event; PCI, percutaneous coronary intervention; CABG, coronary artery bypass graft, SHAP, shapley additive explanations.

The SHAP summary plots depicting the random forest's attributions are represented in Supplementary Figure S2. To illustrate, when examining 1-year cardiovascular death, the SHAP values for BMI range from −0.0009 (minimum) to 0.0287 (maximum), with a median value of 0.0139. In addition, including the predictor BMI in the random forest model resulted in a 1.39% increase in the probability of experiencing cardiovascular death during the 1-year follow-up, particularly for a participant at the median level. Interestingly, the impact of BMI exhibited a varied pattern across different outcomes, elucidated through the SHAP dependence plots showcased in Figure 4. For instance, in the context of 3-year MACE (Figure 4F), as the BMI value increases, its corresponding SHAP value traces a U-shaped trajectory, signifying a sequence of decline followed by elevation. This distinctive pattern is exemplified by the interplay between blue and red dots representing low and high C-reactive protein levels, respectively. Additionally, a noticeable correlation emerges between BMI and C-reactive protein levels in terms of their SHAP values for MACE at the 3-year follow-up.

## 4 Discussion

**Figure 5:**
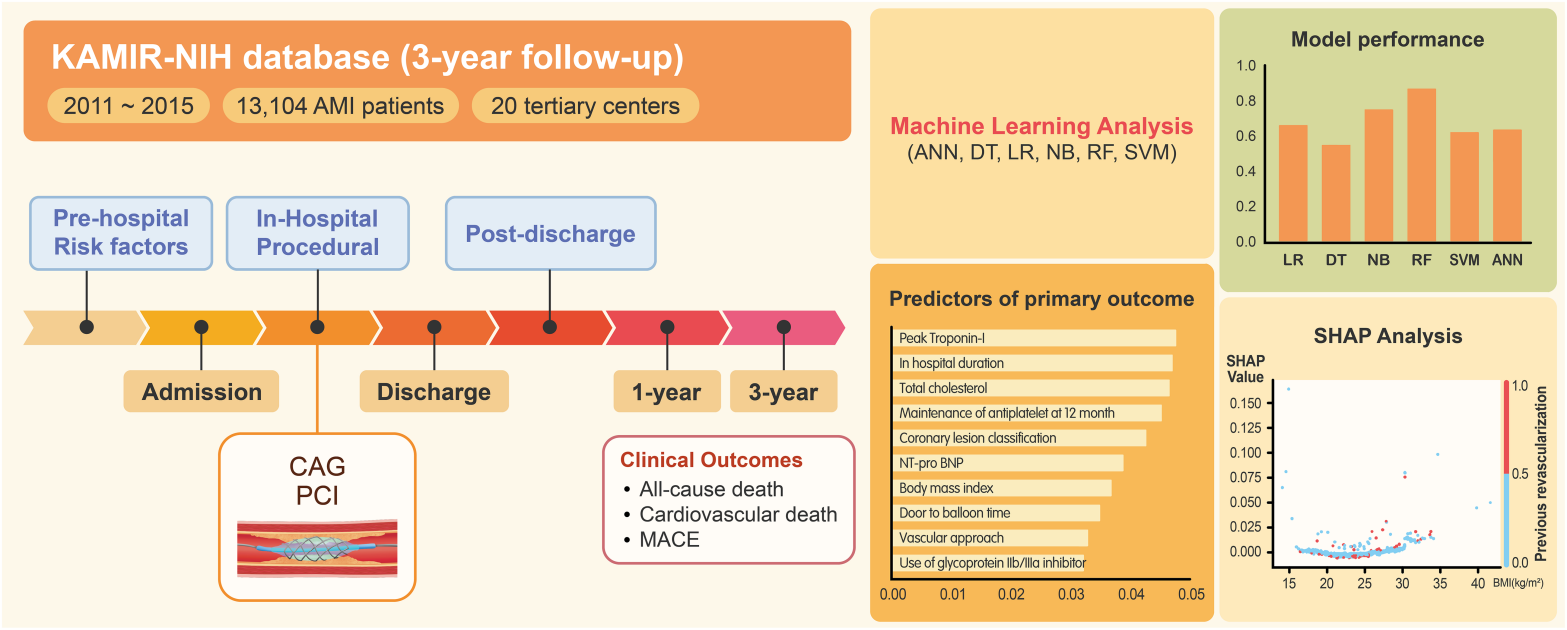
Overview of machine learning analysis of clinical outcomes after AMI. Based on a nationwide AMI registry from Korea, machine learning analysis was performed to predict clinical outcomes after AMI. The analysis encompassed a wide range of patient-related factors, spanning from prehospitalization to postdischarge variables (Left). The random forest algorithm demonstrated the best predictive performance (Right, top). The identified predictors demonstrated nonlinear, variable influences (SHAP analysis) that reflected the complex interplay of multiple factors in real-world scenarios (Right, bottom). AMI, acute myocardial infarction; KAMIR-NIH, Korean acute myocardial infarction-national institutes of health; ANN, artificial neural network; DT, decision tree; LR, logistic regression; NB, naïve Bayes; RF, random forest; SVM, support vector machine; MACE, major adverse cardiovascular event; CAG, coronary angiography; PCI, percutaneous coronary intervention; SHAP, shapley additive explanations; NT-proBNP, N-terminal pro-brain natriuretic peptide.

Based on a nationwide prospective registry of AMI patients, we developed a machine learning-based prediction model that estimates major long-term outcomes after AMI. The main findings from this study could be summarized as follows (Figure 5). First, the machine learning-based algorithms, especially the random forest approach, outperformed the conventional logistic regression-based algorithms in predicting the outcomes after AMI. In particular, the random forest model's prediction of the primary and secondary outcomes of cardiovascular death at 1-year resulted in outstanding performance (with AUC values of 0.874 and 0.881, respectively). Second, diverse patient-related factors from different time periods were included to predict outcomes. The factors used were (1) prehospital variables (demographic factors and comorbidities), (2) in-hospital variables (laboratory markers and clinical events during index admission), (3) procedure-related variables (coronary lesion severity and revascularization strategies), and (4) postdischarge variables (medications and patient compliance). Lastly, the identified predictors did not influence the outcomes in a uniform, linear manner, as typically seen with conventional regression-based algorithms. The results were nonlinear, exerting variable influences as indicated by the SHAP values. These values effectively captured the amalgamation of factors in the real-world scenario, reflecting the inherent outcome variability.

### 4.1 Predictors of cardiovascular outcomes after AMI

The predictors of the 1-year and 3-year outcomes did not reflect significant differences in their characteristics. Specifically, the prehospital, in-hospital, postdischarge, and procedural factors demonstrated close associations with both the 1-year and 3-year outcomes following AMI. Among these risk factors spanning different time periods, the in-hospital risk factors and procedure-related factors accounted for approximately two-thirds of the top 30 predictors for all outcomes. This trend was even more pronounced in the context of the 3-year outcomes. Notably, maintenance of statin therapy at discharge emerged as the most pivotal predictor of the 3-year outcome. This finding implies that after acute revascularization of coronary lesions, the maintenance of potent lipid-lowering therapies becomes a crucial therapeutic strategy that significantly impacts long-term outcomes after AMI. Furthermore, among the various predictors, BMI was identified as one of the most powerful predictors of outcomes after AMI. However, BMI did not exert a uniform influence on the outcomes. This phenomenon is further discussed in detail in the following.

### 4.2 Prediction models in AMI

Several predictive models to assess mortality after AMI have been introduced in the past two decades, and most have been based on conventional regression-based algorithms (7, 8, 20). Among these, the thrombolysis in the myocardial infarction (TIMI) risk score and global registry of acute coronary events (GRACE) risk score have emerged as robust prediction scores (7, 20). These scores were formulated during the early phase of development of PCI and are tailored to predict short-term mortality (30 days to 6 months) after AMI. They encompass simple predictors of in-hospital variables, such as age, blood pressure, and electrocardiographic findings. Both of these scores were constructed using substantial populations of AMI cases during an earlier period and subsequently validated successfully with validation cohorts. However, the predictive performances of the TIMI and GRACE risk scores are relatively low (with AUC values of 0.746 and 0.75, respectively), and their applicability may be limited due to the evolution of therapeutic strategies for coronary revascularization and medical therapy.

Recently, several prediction models employing machine learning algorithms have been developed to predict mortality in AMI patients. One machine learning-based model, utilizing data from the previous KAMIR registry (2006–2013), exhibited a fair discriminative power (AUC 0.915) in predicting 1-year mortality after AMI (8). This model incorporated both in-hospital and procedural variables as predictor candidates. In addition, it also reflected unmodifiable risk factors or initial clinical factors as important predictors of 1-year mortality (such as age, left ventricular ejection fraction, initial Killip class, BMI, initial creatinine, and low-density lipoprotein levels). Similarly, a more recent machine learning-based risk stratification model (the PRAISE score) also demonstrated remarkable performance in predicting 1-year all-cause death after acute coronary syndrome (AUC 0.82 for internal validation, 0.92 for external validation) (21). The PRAISE score encompasses eight predictors of 1-year all-cause death, including left ventricular ejection fraction, age, hemoglobin level, statin use at discharge, estimated glomerular filtration rate, use of angiotensin-converting enzyme inhibitor or angiotensin-II receptor blockers at discharge, previous bleeding, and malignancy. While the specific variables investigated may differ from those used in our study, our prediction model aligns with the PRAISE score in two key aspects. Both models incorporate (i) initial in-hospital variables that reflect hypoperfusion (left ventricular ejection fraction and creatinine and hemoglobin levels) and (ii) postdischarge medications (statin use at discharge) as important predictors of 1-year all-cause death.

### 4.3 New paradigm on the obesity paradox in AMI

We investigated the relative influence of BMI on different outcomes using SHAP dependence plots. In this study, we demonstrated that BMI was an important predictor of all outcomes, with this factor consistently ranking within the top seven predictors, except for 3-year MACE. However, the SHAP dependence plot has revealed varying effects of BMI on each outcome, contingent on its value. For example, the degree of obesity represented by BMI did not significantly impact 1-year all-cause mortality or 1-year cardiovascular mortality. However, it did result in a U-shaped influence on 1-year and 3-year MACE and all-cause mortality. Additionally, we explored the interplay between BMI and outcomes in the presence of other independent variables. For example, the absence of new-onset heart failure during index admission exhibited a strong association with BMI in the context of 1-year all-cause mortality, while a low level of C-reactive protein showed a similar association with BMI in the context of 3-year MACE.

The relative influence described by the SHAP dependence plots partly aligns with the obesity paradox observed in coronary heart disease and heart failure, However, it enhances the existing paradigm by highlighting several key points: (i) obesity assessed through BMI does not affect various cardiovascular outcomes in a uniform manner; (ii) the degree of obesity exerts varying degrees of influence on outcomes; and (iii) obesity is also associated with other independent variables (22–25). The nonlinear and dynamic impacts of BMI on different cardiovascular outcomes complement the observed obesity paradox in cardiovascular contexts (24, 26). Thus, this implies the emergence of a new paradigm that reflects the uncertainties inherent in the real world that cannot be fully explained by conventional statistics.

In this study, we investigated a validated, multi-center registry of AMI that includes more than 9,000 patients and developed machine learning-based algorithms that could predict various long-term outcomes. Large volumes of clinical variables that rise from different times were also included. We included extensive clinical variables spanning various time points and scrutinized their correlations with distinct clinical outcomes. In comparison to conventional regression-based methods, our machine learning-based prediction algorithms showcased superior discriminatory capabilities across all outcomes. To the best of our knowledge, this is the first study to investigate the dynamic interplay of individual predictors across varying degrees, influencing diverse outcomes. Moreover, we delved into the associated covariates that mirrored the intricacies, variations, and uncertainties of the real-world scenario among patients with AMI.

### 4.4 Limitations

This study has several limitations. First, while the original registry reported a 1-year mortality of 4.3%, our cohort exhibited a considerably lower incidence of 1-year mortality (0.4%). This discrepancy could be attributed to the exclusion of variables with missing values. Notably, individuals who did not survive across various time intervals may have been associated with a higher likelihood of missing data. Consequently, this approach might have introduced a selection bias, shifting the patient cohort towards those with milder conditions. Second, the current prediction model has not been externally validated. Although the training set included more than 7,000 patients that was internally validated, validation with independent datasets may improve the generalizability of our prediction model. Third, investigating the variety of mechanisms that could exist between the six dependent variables and their major predictors was beyond the scope of this study. Limited attention has been devoted to this aspect, underscoring the need for further exploration in this direction. Fourth, regarding the rapidly evolving interventional strategy and change of the clinical guidelines, prediction model based on relatively remote data may need to be updated with recent data. Nonetheless, KAMIR-NIH registry is a validated, nationwide registry of AMI, and independent variables included in our database are risk factors that still matters in clinical field. Lastly, synthesizing diverse modes of machine learning-based methods for different types of cardiovascular data could pioneer a new approach in this field.

## 5 Conclusion

In AMI patients, numerous patient-related risk factors spanning the prehospitalization, in-hospitalization, and postdischarge periods exert an impact on both 1-year and 3-year outcomes. Dynamic associations among risk factors should be understood, and appropriate clinical interventions are needed after AMI. Our study suggests potential role of machine learning-based algorithm to predict adverse outcomes in patients with AMI. Further validation based on an external cohort may be needed to generalize the prediction model.

## Data Availability

The original contributions presented in the study are included in the article/**Supplementary Material**, further inquiries can be directed to the corresponding authors.
