# Supplementary material for: Prediction of longitudinal clinical outcomes after acute myocardial infarction using a dynamic machine learning algorithm
Source: Front Cardiovasc Med. 2024 Apr 5;11:1340022. doi: 10.3389/fcvm.2024.1340022 (PMC11027893; doi:10.3389/fcvm.2024.1340022)
Supplement: Supplementary file 1 [file Datasheet1.docx]

**Supplementary Materials**

**Supplementary Table 1. Definition of independent variables**

| **No.** | **Variables** | **Definition** |
| --- | --- | --- |
| 1 | Age | Age (years) at date of admission |
| 2 | Anterior myocardial infarction | Location of myocardial infarction localized by 12-lead electrocardiography |
| 3 | Atrial fibrillation | Atrial fibrillation detected by 12-lead electrocardiography during admission |
| 4 | Atrioventricular block | Presence of 2nd or higher degree of atrioventricular block at initial 12-lead electrocardiography |
| 5 | Beta-blocker at discharge | Use of beta-blocker at discharge |
| 6 | Body mass index | Body mass index (kg/m2) at the date of admission |
| 7 | Cardiogenic shock | Presence of cariogenic shock at either initial presentation or during admission, defined as: systolic blood pressure <90 mm Hg for more than 30 minutes or the need for supportive management to maintain systolic blood pressure>90mmHg; clinical signs of pulmonary congestion; and evidence of impaired end-organ perfusion with at least 1 of the following: cool extremities, decreased urine output, increased lactic acid level, or altered mental status |
| 8 | Cerebrovascular complication | Cerebrovascular complication composite of ischemic stroke or hemorrhagic stroke during admission |
| 9 | Coronary complication | Coronary complication composite of recurred ischemia, recurred infarct or stent thrombosis during admission |
| 10 | Coronary lesion classification | Morphology of coronary artery lesion type was determined according to the ACC/AHA classification (A/B1/B2/C) |
| 11 | C-reactive protein | High sensitivity C-reactive protein (mg/L) measured at initial admission |
| 12 | Creatinine | Baseline creatinine (mg/dL) measured at initial presentation |
| 13 | Culprit lesion | Culprit lesion among (1) left main artery, (2) left anterior descending artery, (3) left circumflex artery, or (4) right coronary artery |
| 14 | Diabetes mellitus | Previous medical history of diabetes mellitus |
| 15 | Diastolic blood pressure | Initial diastolic blood pressure (mmHg) measured at the date of admission |
| 16 | Door to balloon time | Duration of hours from hospital arrival to balloon angioplasty |
| 17 | Dyslipidemia | Previous medical history of dyslipidemia |
| 18 | Dyspnea | Presence of dyspnea at initial presentation |
| 19 | Family history of coronary artery disease | Family history of coronary artery disease |
| 20 | First medical contact | First medical contact prior to initial presentation: (1) emergency medical service, (2) transfer from other hospital, (3) during admission |
| 21 | HbA1c | HbA1c (%) measured during admission, regardless of diabetes mellitus |
| 22 | Heart rate | Initial heart rate measured at day of admission |
| 23 | Hemoglobin | Baseline hemoglobin (g/dL) measured at initial presentation |
| 24 | High-intensity statin at discharge | High-intensity statin defined as by 2013 American College of Cardiology and American Heart Association guidelines: (1) atorvastatin 40mg or more, or (2) rosuvastatin 20mg or more |
| 25 | History of antiplatelet use | Use of antiplatelet medication before admission |
| 26 | Hypertension | Previous medical history of hypertension |
| 27 | In hospital duration | Duration of days from initial admission to discharge |
| 28 | Initial cardiac arrest | Presence of cardiac arrest at initial presentation (regardless of rhythm) |
| 29 | Initial TIMI flow of culprit lesion | Initial TIMI flow of culprit lesion from 0 to 3 |
| 30 | Killip class | Killip classification of acute myocardial infarction (I to IV) at initial presentation |
| 31 | Left ventricular ejection fraction | Left ventricular ejection fraction (%) determined by transthoracic echocardiography at the time of admission, by modified Simpson's biplane method |
| 32 | Left ventricular end diastolic dimension | Left ventricular end diastolic dimension (mm) determined by transthoracic echocardiography at the time of admission |
| 33 | Low-density lipoprotein | Low-density lipoprotein (mmol/L) measured after 8 hours of fasting |
| 34 | MACE at 6 months | Occurrence of MACE (composite outcome of (1) MI, (2) repeat PCI of both target and non-target vessel revascularization, (3) CABG, (4) stent thrombosis, (5) cerebrovascular accident [ischemic stroke or hemorrhagic stroke], and (6) re-hospitalization due to heart failure aggravation) at 6 month follow up |
| 35 | Maintenance of antiplatelet at 12 months | Number of antiplatelet maintained at 12 month follow up: (1) Dual-antiplatelet, (2) Monotherapy of aspirin or P2Y12 inhibitor, (3) None, (4) Unknown |
| 36 | Maintenance of beta-blocker at 12 months | Maintenance of beta-blocker at 12 month follow up: (1) Maintained, (2) Not maintained, (3) Unknown |
| 37 | Maintenance of renin-angiotensin system inhibitor at 12 months | Maintenance of renin-angiotensin system inhibitor (either angiotensin-converting enzyme inhibitor or angiotensin II receptor blocker) at 12 month follow up: (1) Maintained, (2) Not maintained, (3) Unknown |
| 38 | Maintenance of statin at 12 months | Maintenance of statin at 12 month follow up: (1) Maintained, (2) Not maintained, (3) Unknown |
| 39 | New onset heart failure | Clinical evidence of heart failure during admission, without previous history of heart failure |
| 40 | Non-cardiovascular complication | Non-cardiovascular complication, composite of (1) major bleeding (resulting decrease of hemoglobin [≥ 5.0 g/dL]), (2) multi-organ failure, and (3) sepsis |
| 41 | NT-proBNP | Baseline NT-proBNP (pg/mL) measured at initial presentation |
| 42 | Number of stents | Total number of stents implanted in both IRA and non-IRA |
| 43 | Onset to balloon time | Duration of hours from initial onset of symptom to balloon angioplasty |
| 44 | Onset to door time | Duration of hours from initial onset of symptom to hospital arrival |
| 45 | P2Y12 inhibitor at discharge | Type of P2Y12inhibitor prescribed at discharge: (1) Clopidogrel, (2) Prasugrel, (3) Ticagrelor, or (4) None |
| 46 | Peak CK-MB | The highest CK-MB level (ng/mL) measured during admission |
| 47 | Peak Troponin-I | The highest Troponin-I level (ng/mL) measured during admission |
| 48 | Post PCI TIMI | Final TIMI flow of culprit lesion after PCI |
| 49 | Previous history of cerebrovascular accident | Previous history of cerebrovascular accident, including ischemic stroke, hemorrhagic stroke, and transient ischemic attack |
| 50 | Previous history of heart failure | Previous clinical history of heart failure evaluated by echocardiography |
| 51 | Previous history of myocardial infarction or angina | Previous clinical history of myocardial infarction or angina |
| 52 | Previous history of PCI or CABG | Previous history of PCI (at any artery) or history of CABG |
| 53 | Renin-angiotensin system inhibitor at discharge | Use of renin-angiotensin system inhibitor at discharge |
| 54 | Regional Wall Motion Index | Regional Wall Motion Index determined by transthoracic echocardiography at the time of admission |
| 55 | Rhythm disturbance complication | Complication related to rhythm disturbance, composite of atrioventricular block, ventricular tachycardia, or ventricular fibrillation |
| 56 | Sex | Male or Female |
| 57 | Smoking status | Smoking status classified as (1) Current smoker, (2) Ex-smoker, (3) Never smoker, and (4) Unknown |
| 58 | Staged PCI | Staged PCI (Delayed PCI or stepwise PCI) or single-stage PCI |
| 59 | Statin at discharge | Use of statin at discharge |
| 60 | STEMI | Final clinical and angiographic evidence of STEMI |
| 61 | Stent diameter | Diameter of stent (mm) implanted at IRA |
| 62 | Stent generation | Generation of stent implanted at IRA: (1) Bare metal stent, (2) First-generation drug-eluting stent, (3) Second-generation drug-eluting stent, (4) Balloon angioplasty or none |
| 63 | Stent or balloon | Treatment strategy at IRA: (1) Stent implant, (2) Balloon angioplasty, or (3) None |
| 64 | Systolic blood pressure | Initial systolic blood pressure (mmHg) measured at the date of admission |
| 65 | Thrombus aspiration | Thrombus aspiration performed during procedure |
| 66 | Total cholesterol | Total cholesterol (mmol/L) measured after 8 hours of fasting |
| 67 | Total Stent length | Total length (mm) of stent implanted in both IRA and non-IRA |
| 68 | Triglyceride | Triglyceride (mmol/L) measured after 8 hours of fasting |
| 69 | Typical chest pain | Typical chest pain consists of three criteria of (1) substernal chest pain, (2) that is provoked by exertion, and (3) relieved by rest or nitroglycerine |
| 70 | Use of ECMO | Use of ECMO during admission |
| 71 | Use of glycoprotein IIb/IIIa inhibitor | Use of glycoprotein IIb/IIIa inhibitor of Abciximab or Tirofiban during procedure |
| 72 | Use of IABP | Use of IABP at during admission |
| 73 | Use of insulin | Use of insulin at initial admission |
| 74 | Use of IVUS or OCT | Use of IVUS or OCT during procedure |
| 75 | Vascular approach (Transfemoral/Radial) | Vascular approach (Transradial or transfemoral) for PCI |
| 76 | Ventricular tachycardia or ventricular fibrillation | Occurrence of ventricular tachycardia or ventricular fibrillation during admission |
| 77 | Weekend admission | Admission at weekend (Saturday, Sunday) |

BMI, body mass index; HbA1c, glycosylated hemoglobin type A1c; LDL, low density lipoprotein; ECMO, extra-corporeal membrane oxygenation; ECPR, extracorporeal cardiopulmonary resuscitation; STEMI, ST-elevation myocardial infarction; TIMI, thrombolysis in myocardial infarction; CABG, coronary artery bypass graft; IABP, Intra-aortic balloon pump; IRA, infarct-related artery; CRRT, Continuous renal replacement therapy; LV EF, left ventricular ejection fraction; Hb, hemoglobin; CK-MB, creatine kinase-MB; NT-proBNP, N-terminal prohormone of brain natriuretic peptide.

**Supplementary Table 2. Baseline characteristics of study participants for 3-year outcome**

| **Variables** | **Total (n=8,806)** |
| --- | --- |
| **Pre-hospital variables** |  |
| Age, years | 62.2 ± 12.0 |
| Female sex | 2,036 (23.1) |
| Body mass index (kg/m2) | 24.2 ± 3.1 |
| Smoking status |  |
| Current smoker | 3,795 (43.1) |
| Hypertension | 4,267 (48.5) |
| Diabetes mellitus | 2,270 (25.8) |
| HbA1c (%) | 6.2 ± 1.2 |
| Dyslipidemia | 1,047 (11.9) |
| Baseline low-density lipoprotein (mg/dL) | 114.3 ± 37.0 |
| Baseline high-density lipoprotein (mg/dL) | 42.6 ± 11.1 |
| Baseline triglyceride (mg/dL) | 135.4 ± 111.8 |
| Baseline total cholesterol (mg/dL) | 181.3 ± 43.8 |
| Previous history of myocardial infarction or angina | 1,169 (13.3) |
| Previous history of PCI or CABG | 847 (9.6) |
| Previous history of heart failure | 76 (0.9) |
| Previous history of cerebrovascular accident | 510 (5.8) |
| Family history of coronary artery disease | 625 (7.1) |
| **In-hospital variables** |  |
| Typical chest pain | 7,913 (89.9) |
| Dyspnea | 1,750 (19.9) |
| Killip class |  |
| I | 7,314 (83.1) |
| II | 670 (7.6) |
| III | 464 (5.3) |
| IV | 358 (4.1) |
| STEMI | 4,558 (51.8) |
| Anterior myocardial infarction | 3,361 (38.2) |
| Atrioventricular block (2nd degree or more) | 74 (0.8) |
| Atrial fibrillation | 229 (2.6) |
| Ventricular tachycardia or ventricular fibrillation | 328 (3.7) |
| New onset heart failure | 246 (2.8) |
| Initial cardiac arrest | 396 (4.5) |
| Cardiogenic shock | 514 (5.8) |
| Use of extracorporeal membrane oxygenation | 23 (0.3) |
| Systolic blood pressure (mmHg) | 131.1 ± 28.3 |
| Diastolic blood pressure (mmHg) | 79.5 ± 17.4 |
| Heart rate | 77.2 ± 17.9 |
| Peak creatine-kinase myoglobin (ng/mL) | 113.9 ± 145.0 |
| Peak Troponin-I (ng/mL) | 43.7 ± 98.9 |
| NT-proBNP (pg/mL) | 1016.6 ± 4845.1 |
| Hemoglobin (g/dL) | 14.1 ± 1.9 |
| Creatinine (mg/dL) | 1.0 ± 0.8 |
| C-reactive protein (mg/L) | 0.7 ± 2.7 |
| Left ventricular ejection fraction (%) | 52.8 ± 10.1 |
| Regional wall motion index | 1.3 ± 0.3 |
| In hospital duration (days) | 4.9 ± 4.3 |
| **Procedural variables** |  |
| Onset to door time (hours) | 21.3 ± 70.1 |
| Door to balloon time (hours) | 19.7 ± 428.0 |
| Onset to balloon time (hours) | 41.0 ± 434.8 |
| Vascular approach (Transfemoral/Radial |  |
| Transfemoral | 5,347 (60.7) |
| Transradial | 3,361 (38.2) |
| Both | 98 (1.1) |
| Culprit lesion |  |
| Left anterior descending | 4,113 (46.7) |
| Left circumflex | 1,549 (17.6) |
| Right coronary artery | 2,988 (33.9) |
| Left main | 156 (1.8) |
| Coronary lesion classification |  |
| A | 115 (1.3) |
| B1 | 1,033 (1.7) |
| B2 | 3,363 (38.2) |
| C | 4,295 (48.8) |
| Initial TIMI flow of culprit lesion |  |
| 0 | 4,137 (47.0) |
| 1 | 976 (11.1) |
| 2 | 1,379 (15.7) |
| 3 | 2,314 (26.3) |
| Post PCI TIMI |  |
| 0 | 22 (0.2) |
| 1 | 23 (0.3) |
| 2 | 195 (2.2) |
| 3 | 8,566 (97.3) |
| Stent or balloon |  |
| None | 27 (0.3) |
| Stent | 8,232 (93.5) |
| Balloon | 547 (6.2) |
| Number of stents | 1.3 ± 0.8 |
| Total Stent length (mm) | 27.4 ± 15.4 |
| Stent generation |  |
| No stent | 574 (6.5) |
| Bare metal stent | 196 (2.2) |
| 1st generation | 712 (8.1) |
| 2nd generation | 7,324 (83.2) |
| Staged PCI | 869 (9.9) |
| Thrombus aspiration | 2,219 (25.2) |
| Use of glycoprotein IIb/IIIa inhibitor | 1,386 (15.7) |
| Use of intra-aortic balloon pump | 168 (1.9) |
| Use of IVUS or OCT | 1,956 (22.2) |
| **Post-discharge variables** |  |
| P2Y12 inhibitor at discharge |  |
| None | 71 (0.8) |
| Clopidogrel | 5,957 (67.6) |
| Prasugrel | 1,020 (11.6) |
| Ticagrelor | 1,758 (20.0) |
| Beta-blocker at discharge | 7,695 (87.4) |
| Renin-angiotensin system inhibitor at discharge | 7,332 (83.3) |
| Statin at discharge | 8,416 (95.6) |
| Use of high-intensity statin at discharge | 2,528 (28.7) |
| MACE at 6 months | 291 (3.3) |
| Maintenance of antiplatelet at 12 months |  |
| Dual antiplatelet | 6,282 (71.3) |
| Mono antiplatelet | 2,413 (27.4) |
| No antiplatelet | 111 (1.3) |
| Maintenance of beta-blocker at 12 months | 7,043 (80.0) |
| Maintenance of renin-angiotensin system inhibitor at 12 months | 6,632 (75.3) |
| Maintenance of statin at 12 months | 8,294 (94.2) |
| **Outcomes** |  |
| All-cause death | 144 (1.6) |
| Cardiovascular death | 79 (0.9) |
| MACE | 254 (2.9) |

NT-proBNP, N-Terminal pro-Brain Natriuretic Peptide; PCI, percutaneous coronary intervention; CABG, coronary artery bypass graft; STEMI, ST-elevation myocardial infarction; MACE, major adverse cardiovascular event; TIMI, thrombolysis in myocardial infarction; IVUS, intravascular ultrasound; OCT, optical coherence tomography.

.

**Supplementary Table 3. Random Forest Variable Importance at 1-year follow-up**

| **VI-Rank** | **Cardiovascular death at 1-year** | | **All-cause death at 1-year** | | **MACE at 1-year** | |  |
| --- | --- | --- | --- | --- | --- | --- | --- |
|  | **Variable** | **VI-Value** | **Variable** | **VI-Value** | **Variable** | **VI-Value** | |
| 1 | Maintenance of antiplatelet at 12 months | 0.0608 | Peak Troponin-I | 0.0477 | In hospital duration | 0.0454 | |
| 2 | Vascular approach (Transfemoral/Radial) | 0.0511 | In hospital duration | 0.0471 | Triglyceride | 0.0406 | |
| 3 | NT-proBNP | 0.0468 | Total cholesterol | 0.0466 | Coronary lesion classification | 0.0371 | |
| 4 | Total cholesterol | 0.0467 | Maintenance of antiplatelet at 12 months | 0.0453 | Use of glycoprotein IIb/IIIa inhibitor | 0.0365 | |
| 5 | Body mass index | 0.0437 | Coronary lesion classification | 0.0427 | Door to balloon time | 0.0360 | |
| 6 | Coronary lesion classification | 0.0372 | NT-proBNP | 0.0389 | C-reactive protein | 0.0356 | |
| 7 | Peak Troponin-I | 0.0368 | Body mass index | 0.0369 | Body mass index | 0.0353 | |
| 8 | Beta-blocker at discharge | 0.0332 | Door to balloon time | 0.0350 | Total cholesterol | 0.0342 | |
| 9 | C-reactive protein | 0.0315 | Vascular approach (Transfemoral/Radial) | 0.0330 | Onset to door time | 0.0336 | |
| 10 | Use of glycoprotein IIb/IIIa inhibitor | 0.0292 | Use of glycoprotein IIb/IIIa inhibitor | 0.0323 | Onset to balloon time | 0.0335 | |
| 11 | Door to balloon time | 0.0287 | Triglyceride | 0.0284 | Peak Troponin-I | 0.0331 | |
| 12 | HbA1c | 0.0280 | Beta-blocker at discharge | 0.0280 | HbA1c | 0.0316 | |
| 13 | In hospital duration | 0.0250 | C-reactive protein | 0.0259 | Low-density lipoprotein | 0.0299 | |
| 14 | Sex | 0.0240 | Family history of coronary artery disease | 0.0248 | Statin at discharge | 0.0295 | |
| 15 | Statin at discharge | 0.0235 | Onset to door time | 0.0242 | NT-proBNP | 0.0295 | |
| 16 | Triglyceride | 0.0232 | Statin at discharge | 0.0240 | Creatinine | 0.0293 | |
| 17 | Onset to door time | 0.0224 | Creatinine | 0.0232 | Sex | 0.0285 | |
| 18 | Culprit lesion | 0.0210 | Sex | 0.0231 | Beta-blocker at discharge | 0.0278 | |
| 19 | Onset to balloon time | 0.0208 | Onset to balloon time | 0.0224 | Family history of coronary artery disease | 0.0275 | |
| 20 | Creatinine | 0.0206 | Total Stent length | 0.0224 | Hemoglobin | 0.0263 | |
| 21 | Maintenance of statin at 12 months | 0.0189 | HbA1c | 0.0217 | Vascular approach (Transfemoral/Radial) | 0.0259 | |
| 22 | Low-density lipoprotein | 0.0188 | Culprit lesion | 0.0211 | Culprit lesion | 0.0254 | |
| 23 | Previous history of myocardial infarction or angina | 0.0188 | Low-density lipoprotein | 0.0210 | Total Stent length | 0.0241 | |
| 24 | Family history of coronary artery disease | 0.0181 | Hemoglobin | 0.0194 | New onset heart failure | 0.0147 | |
| 25 | Hemoglobin | 0.0178 | Maintenance of statin at 12 months | 0.0182 | Number of stents | 0.0129 | |
| 26 | Total Stent length | 0.0158 | Left ventricular ejection fraction | 0.0108 | Staged PCI | 0.0110 | |
| 27 | Previous history of PCI or CABG | 0.0139 | Previous history of myocardial infarction or angina | 0.0106 | Left ventricular end diastolic dimension | 0.0108 | |
| 28 | Maintenance of beta-blocker at 12 months | 0.0105 | MACE at 6 months | 0.0096 | Left ventricular ejection fraction | 0.0105 | |
| 29 | Post PCI TIMI | 0.0099 | Maintenance of beta-blocker at 12 months | 0.0089 | Stent diameter | 0.0087 | |
| 30 | Left ventricular ejection fraction | 0.0092 | Dyslipidemia | 0.0083 | Typical chest pain | 0.0081 | |

MACE, major adverse cardiovascular event; VI, variable importance; NT-proBNP, N-Terminal pro-Brain Natriuretic Peptide; PCI, percutaneous coronary intervention; CABG, coronary artery bypass graft; TIMI, thrombolysis in myocardial infarction.

**Supplementary Table 4. Random Forest Variable Importance at 3-year follow-up**

| **VI-Rank** | **Cardiovascular death at 3-year** | | **All-cause death at 3-year** | | **MACE at 3-year** | |
| --- | --- | --- | --- | --- | --- | --- |
|  | **Variable** | **VI-Value** | **Variable** | **VI-Value** | **Variable** | **VI-Value** |
| 1 | Statin at discharge | 0.0437 | Statin at discharge | 0.0511 | Triglyceride | 0.0414 |
| 2 | Use of glycoprotein IIb/IIIa inhibitor | 0.0431 | Sex | 0.0434 | Use of glycoprotein IIb/IIIa inhibitor | 0.0393 |
| 3 | Body mass index | 0.0429 | Body mass index | 0.0396 | NT-proBNP | 0.0381 |
| 4 | Coronary lesion classification | 0.0390 | Use of glycoprotein IIb/IIIa inhibitor | 0.0382 | HbA1c | 0.0380 |
| 5 | Onset to door time | 0.0389 | In hospital duration | 0.0381 | C-reactive protein | 0.0359 |
| 6 | Peak Troponin-I | 0.0385 | NT-proBNP | 0.0358 | Vascular approach (Transfemoral/Radial) | 0.0354 |
| 7 | Sex | 0.0384 | Coronary lesion classification | 0.0358 | In hospital duration | 0.0352 |
| 8 | Onset to balloon time | 0.0374 | Total cholesterol | 0.0357 | Peak Troponin-I | 0.0343 |
| 9 | HbA1c | 0.0363 | Door to balloon time | 0.0348 | Onset to balloon time | 0.0339 |
| 10 | NT-proBNP | 0.0356 | Peak Troponin-I | 0.0337 | Coronary lesion classification | 0.0339 |
| 11 | C-reactive protein | 0.0344 | C-reactive protein | 0.0335 | Total cholesterol | 0.0338 |
| 12 | In hospital duration | 0.0340 | Onset to balloon time | 0.0331 | Onset to door time | 0.0332 |
| 13 | Low-density lipoprotein | 0.0338 | Onset to door time | 0.0331 | Sex | 0.0331 |
| 14 | Total cholesterol | 0.0325 | HbA1c | 0.0329 | Body mass index | 0.0331 |
| 15 | Door to balloon time | 0.0318 | Low-density lipoprotein | 0.0324 | Door to balloon time | 0.0321 |
| 16 | Triglyceride | 0.0286 | Triglyceride | 0.0306 | Statin at discharge | 0.0310 |
| 17 | Total Stent length | 0.0277 | Creatinine | 0.0271 | Low-density lipoprotein | 0.0293 |
| 18 | Culprit lesion | 0.0264 | Vascular approach (Transfemoral/Radial) | 0.0257 | Creatinine | 0.0288 |
| 19 | Creatinine | 0.0251 | Family history of coronary artery disease | 0.0254 | Beta-blocker at discharge | 0.0272 |
| 20 | Vascular approach (Transfemoral/Radial) | 0.0228 | Hemoglobin | 0.0243 | Family history of coronary artery disease | 0.0255 |
| 21 | Hemoglobin | 0.0217 | Culprit lesion | 0.0238 | Total Stent length | 0.0252 |
| 22 | Family history of coronary artery disease | 0.0193 | Beta-blocker at discharge | 0.0231 | Culprit lesion | 0.0243 |
| 23 | Beta-blocker at discharge | 0.0189 | Total Stent length | 0.0226 | Hemoglobin | 0.0224 |
| 24 | New onset heart failure | 0.0140 | New onset heart failure | 0.0134 | New onset heart failure | 0.0152 |
| 25 | Number of stents | 0.0107 | Staged PCI | 0.0097 | Number of stents | 0.0141 |
| 26 | Left ventricular ejection fraction | 0.0092 | Dyslipidemia | 0.0094 | Left ventricular end diastolic dimension | 0.0106 |
| 27 | P2Y12 inhibitor at discharge | 0.0091 | Number of stents | 0.0094 | Left ventricular ejection fraction | 0.0102 |
| 28 | Left ventricular end diastolic dimension | 0.0091 | Left ventricular ejection fraction | 0.0093 | Staged PCI | 0.0098 |
| 29 | Dyslipidemia | 0.0085 | Stent generation | 0.0088 | MACE at 6 months | 0.0081 |
| 30 | Staged PCI | 0.0082 | Left ventricular end diastolic dimension | 0.0082 | Stent diameter | 0.0077 |

MACE, major adverse cardiovascular event; VI, variable importance; NT-proBNP, N-Terminal pro-Brain Natriuretic Peptide; PCI, percutaneous coronary intervention.

**Supplementary Figure 1. Comparison of performance (Random forest vs. logistic regression)**

**
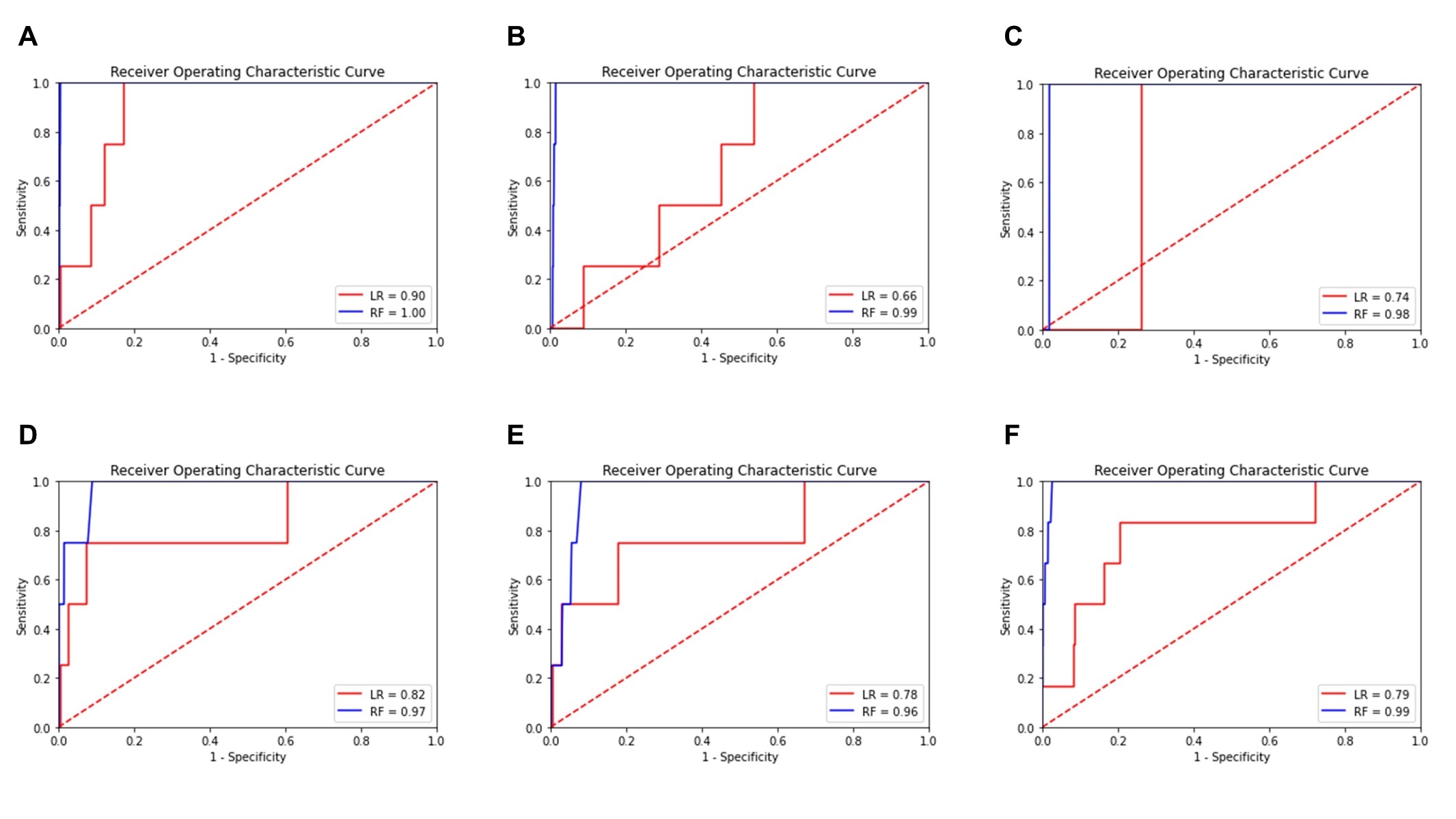
**

The predictive performance of logistic regression and random forest are compared above (Supplementary Figure 1A-F). Note that the area under the receiver-operating-characteristic curve are averaged value of random split and examination (50 times), and graphs above are some examples of them.

LR, logistic regression; RF, random forest.

**Supplementary Figure 2. Random Forest SHAP Summary Plots**

**
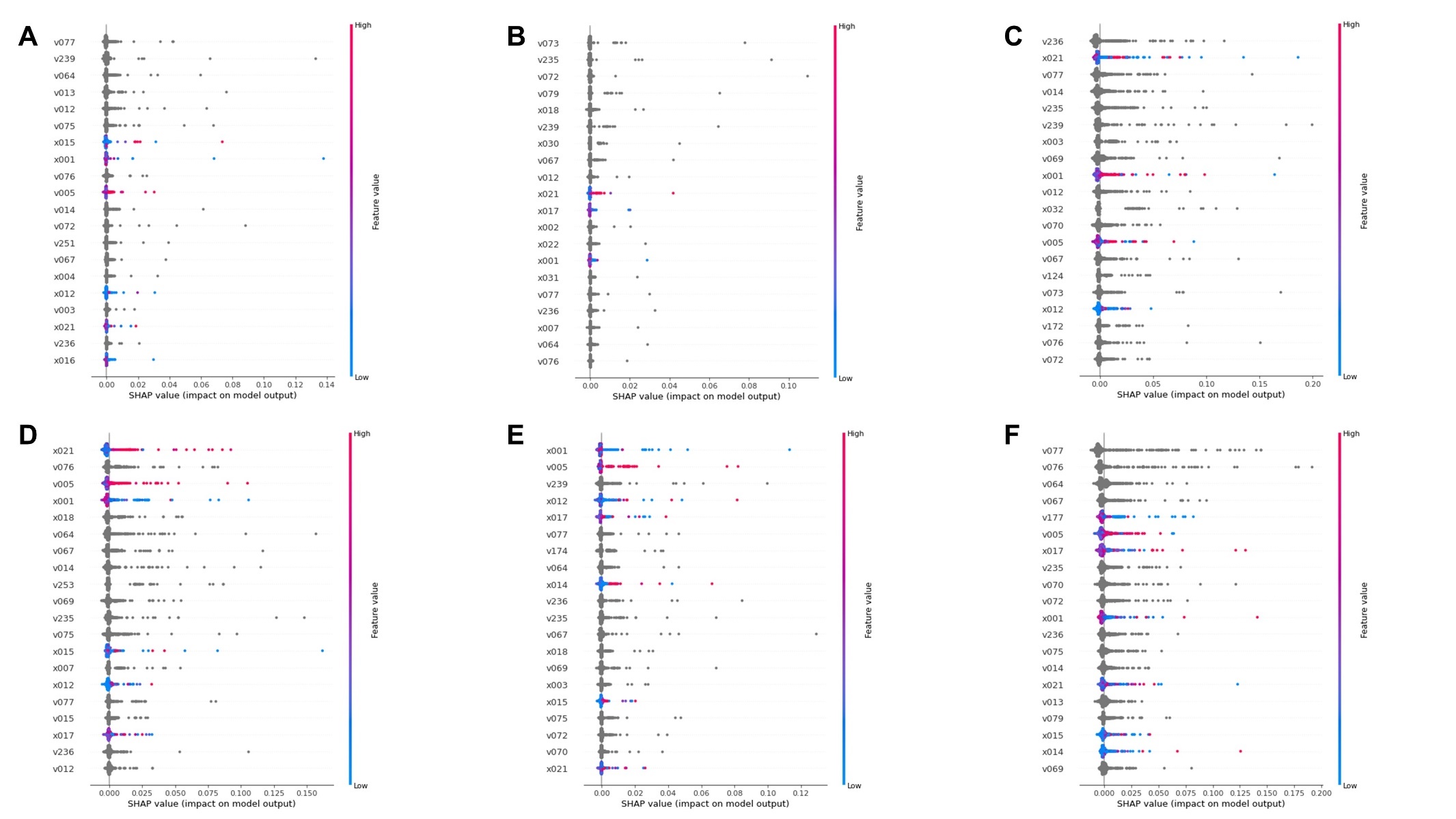
**

**A. All-cause death at 1-year, B. Cardiovascular death at 1-year, C. MACE at 1-year, D. All-cause death at 3-year, E. Cardiovascular death at 3-year, F. MACE at 3-year.**

v003: Sex, v005: Age of onset, v012: Systolic blood pressure, v013: Diastolic blood pressure, v014: Heart rate, v015: Killip class, v064: Hemoglobin, v067: Creatinine, v069: Peak CK-MB, v070: Peak Troponin-I, v072: Total cholesterol, v073: Triglyceride, v075: Low-density lipoprotein, v076: C-reactive protein, v077: NT-proBNP, 079: HbA1c, v124: Beta-blocker at discharge, v172: Coronary lesion classification, v174: Stent or balloon, v177: Stent diameter, v235: Left ventricular ejection fraction, v236: Regional wall motion index, v239: Left ventricular end diastolic dimension, v251: Cardiogenic shock, v253: New onset heart failure, x001: Body mass index, x002: Previous history of myocardial infarction or angina, x003: Previous history of PCI or CABG, x004: Previous history of cerebrovascular accident, x007: Initial cardiac arrest, x012: Onset to door time, x014: Onset to balloon time, x015: Door to balloon time, x016: Number of stents, x017: Total stent length, x018: Stent generation, x021: In hospital duration, x030: Maintenance of antiplatelet at 12 month, x031: Maintenance of statin at 12 month, x032: MACE at 6 month.

HbA1c, glycosylated hemoglobin type A1c; CABG, coronary artery bypass graft; CK-MB, creatine kinase-MB; NT-proBNP, N-terminal prohormone of brain natriuretic peptide, PCI, percutaneous coronary intervention; MACE, major adverse cardiovascular event; TIMI, thrombolysis in myocardial infarction.
